# Computed tomography-based patient-specific cutting guides used for positioning of the femoral component of implants during unicompartmental knee arthroplasty: a cadaver study

**DOI:** 10.1186/s12893-023-02272-4

**Published:** 2023-12-19

**Authors:** Junfeng Cai, Min Ma, Wen Zeng, Shuling Luo, Feng Yuan, Feng Yin

**Affiliations:** Department of joint surgery, Shanghai east hospital, Tongji university, school of medicine, Shanghai, 200120 China

**Keywords:** Component alignment, Femoral cutting guides, Patient-specific instrumentation, Unicompartmental knee arthroplasty

## Abstract

**Background:**

To investigate whether patient-specific instrumentation (PSI) improves the femoral component positioning of implants during unicompartmental knee arthroplasty (UKA) using cadaver bone models.

**Methods:**

Fifty adult cadaveric femoral bone specimens collected from February 2016–2018, were randomized to receive medial UKA with a PSI guide (n = 25) or conventional instrumentation (CI) (n = 25). Standard anteroposterior and lateral view radiographs were obtained postoperatively to assess the coronal and sagittal positioning of the femoral prostheses, respectively. The osteotomy time was recorded to assess the convenience of PSI in guiding osteotomy.

**Results:**

Osteotomy time significantly shortened in the PSI group (3.12 ± 0.65 versus 4.33 ± 0.73 min, p < 0.001). There was a significant difference in the postoperative coronal alignment of the femoral component between the PSI and CI groups (varus/valgus angle: 1.43 ± 0.93° vs. 2.65 ± 1.50°, p = 0.001). The prevalence of outliers in coronal alignment was lower in the PSI than the CI group (2/25, 8% vs. 9/25, 36%). Sagittal posterior slope angle of the femoral component was significantly different between the two groups (8.80 ± 0.65° and 6.29 ± 1.88° in the CI and PSI groups, respectively, p < 0.001). The malalignment rate of the femoral component in the sagittal plane was 60% in the CI group, whereas no positioning deviation was observed in the PSI group.

**Conclusion:**

This study used a cadaver model to support the fact that CT-based PSI shows an advantage over CI in optimizing implant positioning for UKAs.

## Background

Unicompartmental knee arthroplasty (UKA) is the recommended treatment for osteoarthritis of the medial compartment of the knee. Compared to total knee arthroplasty (TKA), UKA can preserve natural knee kinematics and, thus, contribute to a better range of motion, fewer complications, and higher functional scores [1–3].^1–3^ Nonetheless, long-term follow-up studies indicate that the survivorship of UKA prostheses (approximately 85–95%) seems to be lower than that of TKA (at least 90%) subsequently resulting in more revisions (4–32% vs. 2–15%) [4–6]. These findings indicate that improving implant survivorship and reducing revision rates may be an issue that urgently needs to be solved.

Accumulating evidence indicates that malpositioning of the tibial and femoral components is a major contributor to reduced implant longevity [7–10]. Conventionally, a short or long intramedullary guiding rod is used to find the anatomical axis and insertion site on the femoral side of the femoral prosthesis; however, several studies have implied that the use of this method may result in excessive flexion and valgus alignment errors [11–13]. To improve the accuracy of component positioning, the patient-specific instrumentation (PSI) technique has recently been introduced, in which customized cutting guides that match the individual patient’s distal femur are fabricated based on three-dimensional (3D) images obtained from either computed tomography (CT) or magnetic resonance imaging (MRI) [14–16]. Theoretically, the precision of component positioning can be improved with individualized custom cutting guides compared to conventional instrumentation (CI) [17]. This hypothesis was proven by Jaffry et al., who found that femoral implant orientation (6 ± 3^o^ vs. 10 ± 5^o^, p = 0.002) was more accurate in the PSI group than in the CI group [18]. Kerens et al. reported that the ideal alignment for the femoral component was reached in all included patients after the PSI procedure [19]. Femoral compound rotational error was significantly lower in patients in the PSI group than in the CI group (p = 0.004) [20]. However, Alvand et al. found no statistically significant difference in the femoral component positioning of the implants between the PSI and CI groups (varus/valgus angle, 0.9 ± 4.0^o^ versus 1.8 ± 3.0^o^; flexion/extension angle, 9.1 ± 3.0^o^ versus 8.8 ± 4.8^o^; p > 0.05) [21]. Furthermore, Ollivier et al. (frontal angle, 89 ± 2^o^ versus 90 ± 2^o^, p = 0.16; sagittal angle, 10 ± 2^o^ versus 97 ± 2^o^, p = 0.29) [22], Sanz-Ruiz et al. [frontal component angle, 90 (87–92) vs. 88 (86–91), p = 0.38] [23] and Leenders et al. (number of outliers for femoral component frontal plane, 1% versus 0%; femoral component sagittal plane, 8.9% versus 7.1%, p > 0.05) [24] showed that PSI did not have an advantage over CI in improving the positioning of the femoral components. These findings revealed the uncertainty of whether PSI yielded more accurate femoral alignment than CI; therefore, additional comparative experiments are required.

To further confirm the value of PSI in guiding the positioning of the femoral component of implants during UKA, this study aimed (i) to design CT-based individualized cutting guides for cadaver femora for people of Chinese ethnicity preoperatively; (ii) to prospectively compare the convenience of PSI in guiding osteotomy with CI; and (iii) to prospectively explore whether PSI could improve the accuracy of positioning of the femoral component of the implants in comparison with the CI procedure. A meta-analysis on TKA observed that preoperative CT was more beneficial than MRI to produce PSI and reduce the risk of femoral rotational outliers [25]. Therefore, we hypothesized that our study with CT-based PSI (relative to MRI-derived PSI in previous studies [18–24]) may provide robust evidence to demonstrate that PSI could assist in positioning the femoral component more accurately than CI.

## Methods

### Specimens

This study was performed according to Declaration of Helsinki and the protocol has been approved by the ethics committee of our Hospital. Cadaveric specimens were provided by the Department of Anatomy, Medical College of Fudan University, Shanghai, China from February 2016 to February 2018. This anatomical study followed the CACTUS guidelines [26]. The inclusion criteria were as follows: (i) Chinese ethnicity; (ii) adults; and (iii) intact femoral diaphysis regardless of side. The exclusion criteria were as follows: (i) serious lesions in the knee joints, (ii) bone destructive lesions and injuries in the anatomic structures of the bone, and (iii) congenital abnormalities.

Fifty femoral cadaveric specimens of adults (age, 38–75 years; height, 145–178 cm; weight, 38–75 kg) were randomly assigned to receive medial UKA with a patient-specific cutting guide (PSI group, n = 25) or conventional intramedullary guiding rod (CI group, n = 25).

### Preparation of individualized cutting guides

**Fig. 1:**
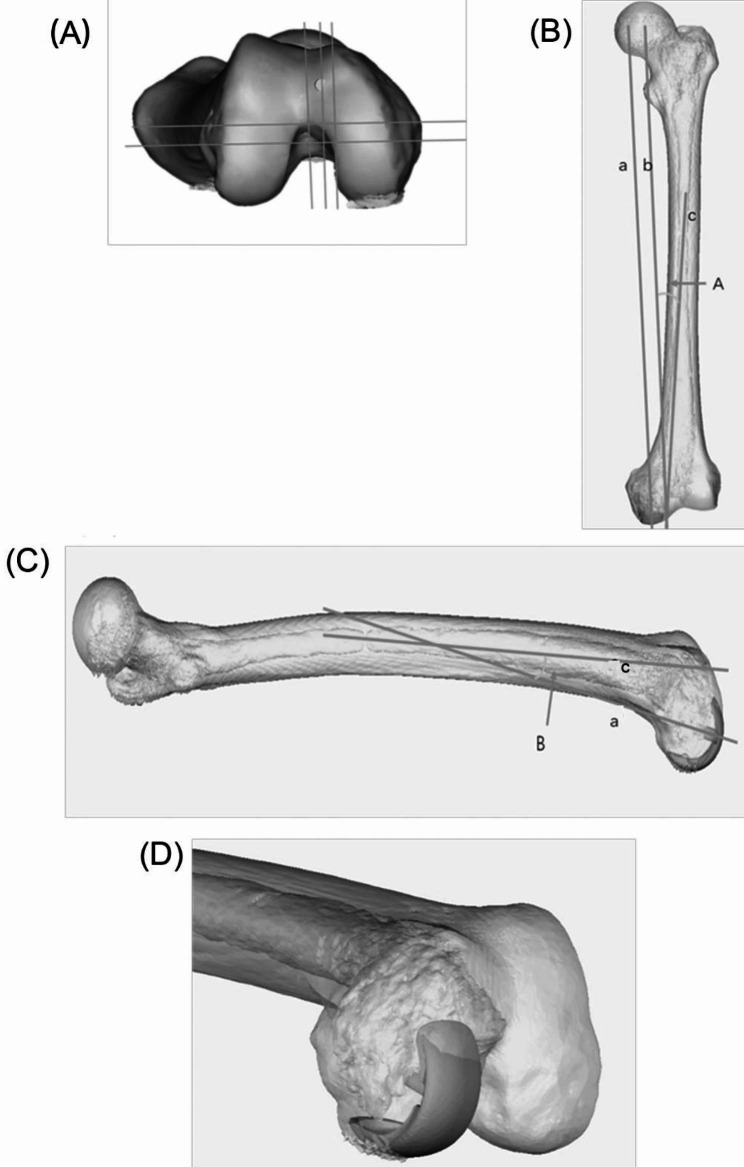
Preparation of individualized cutting guides. (**A**) the line joining the center of the femur and the center of the femoral head was defined as the femoral mechanical axis. (**B**) in the coronal plane, the alignment of prosthesis at the midline of medial condyle of the femur (line a) should be parallel to the femoral mechanical axis (line b). (**C**) in the sagittal plane, the alignment of femoral prosthesis (line a) should be posteriorly sloped to 10^o^ relative to the anatomical axis (line c). (**D**) the optimal prosthesis was selected by simulated installation

The specimens were scanned preoperatively using a Light Speed 16 CT scanner (GE Healthcare Technologies, Wauwatosa, Wisconsin, USA) with a slice thickness of 1.5 mm, speed of 9.37 mm/rotation and a pitch of 0.9 at 120 kV and 200 mA. The CT images were imported into MIMICS software (version 10.0; Materialise NV, Leuven, Belgium) and segmented to generate a 3D model of the femur. This 3D model was loaded into the PTC Creo computer-aided design and manufacturing system (Parametric Technologies Corp., Needham, Massachusetts, USA) to mark feature points and lines. The line joining the center of the femur and the center of the femoral head was defined as the femoral mechanical axis (Fig. 1A). In the coronal plane, alignment of the prosthesis at the midline of the medial condyle of the femur should be parallel to the femoral mechanical axis (Fig. 1B). In the sagittal plane, the alignment of the femoral prosthesis should be sloped posteriorly to 10^o^ relative to the anatomical axis (Fig. 1C). After confirming the alignment of the femoral prosthesis, the optimal prosthesis (Biomet, Warsaw, Indiana, USA) (Fig. 1D) was selected by simulated installation.

**Fig. 2:**
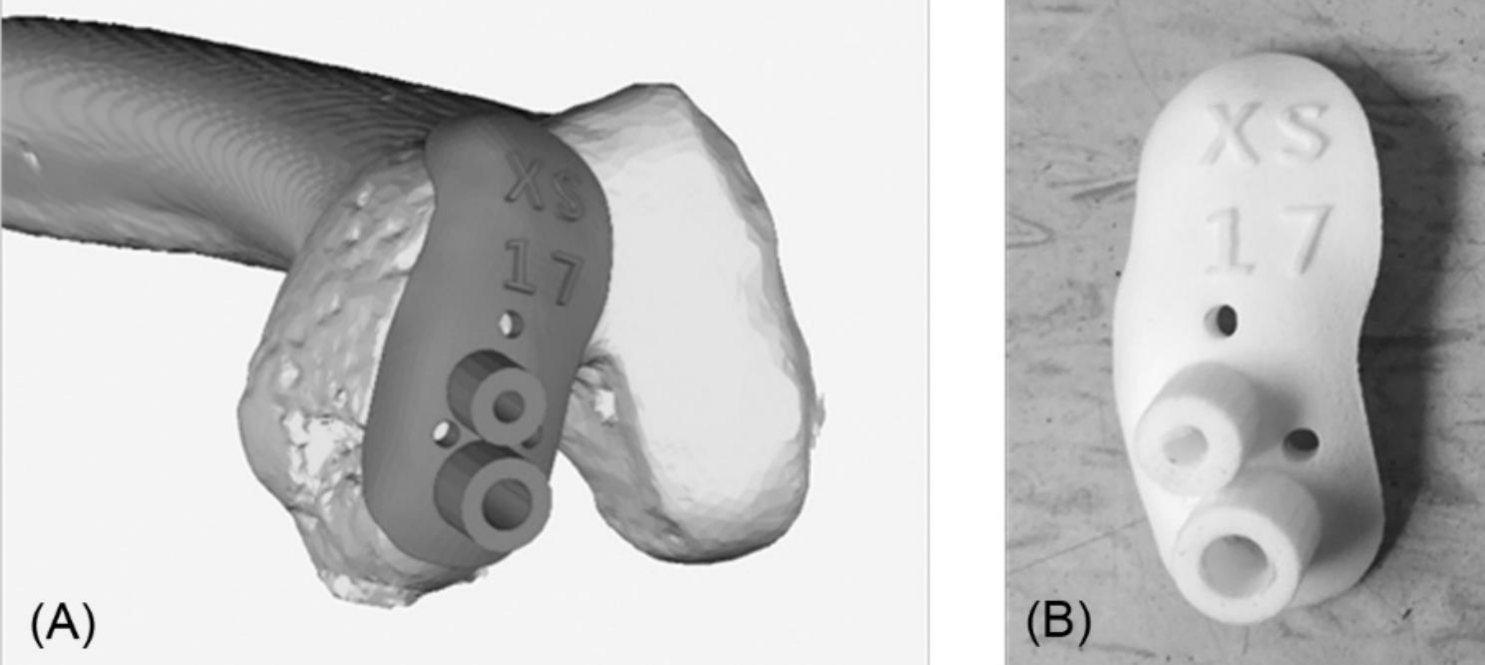
Manufactured patient-specific cutting guides. (**A**) 3D-model, and (**B**) 3D printed cutting guides

According to the planned location of the femoral prosthesis and the corresponding model of the bone cutting tools, the pilot hole positions and orientations of traditional metal bone cutting tools at the femur were automatically determined through the femoral mechanical coordinate system, following which the pilot hole positions, and orientations of the cutting guide were identified. After parametric modeling, the positions, and orientations of pilot holes in the prosthesis were parameterized, which were then combined with the morphology of the femoral condyle to prepare individualized guide plates. The Boolean function was used to integrate the positions and orientations of the pilot holes in the prosthesis and guide plates based on morphological characteristics to fabricate individualized cutting guides for the femur. A 3D cutting guide model was produced using a selective laser sintering rapid prototyping machine with nylon as the selective laser sintering substrate (Fig. 2).

### Surgical procedure

**Fig. 3:**
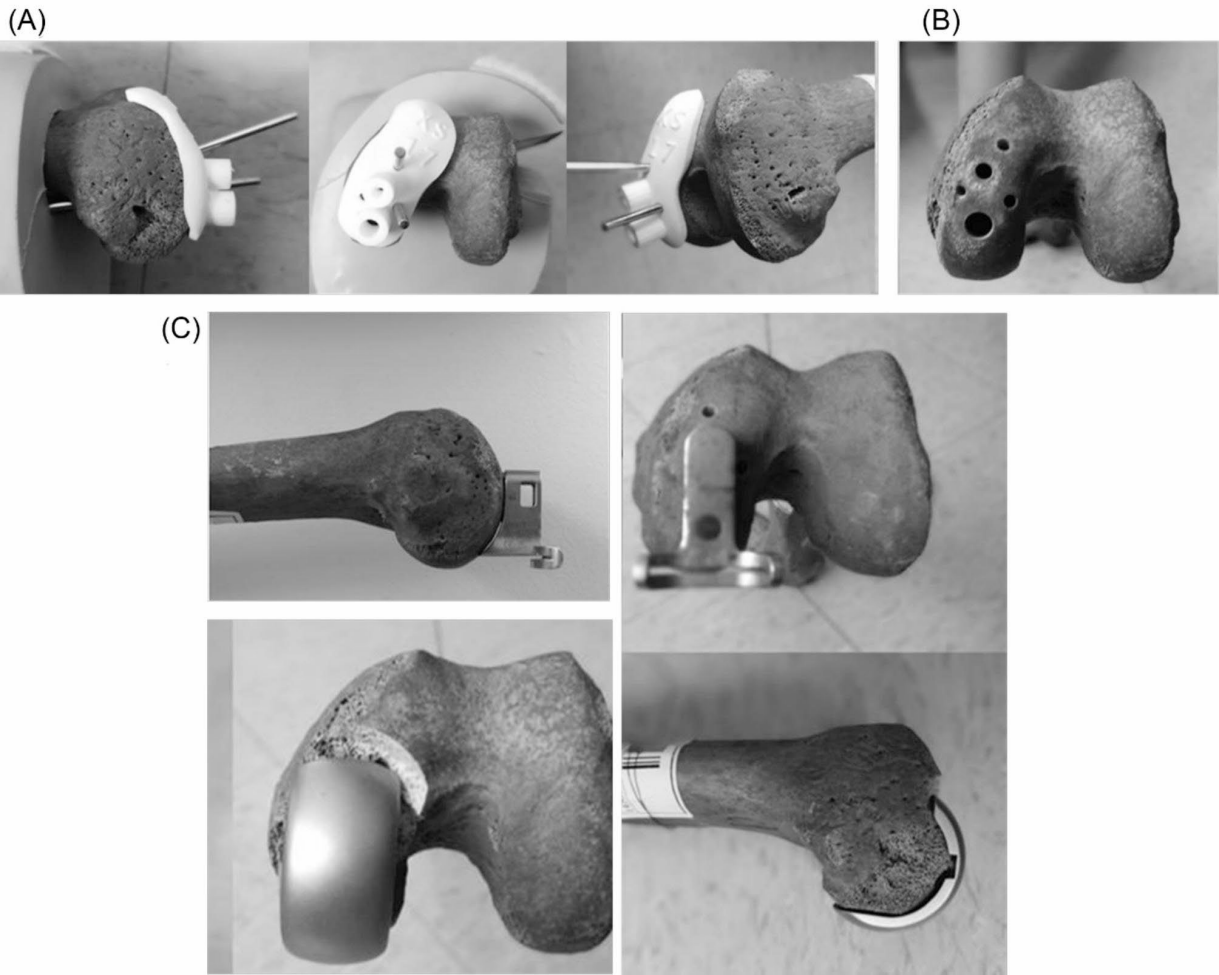
The application of patient-specific cutting guides for medial unicompartmental knee arthroplasty. (**A**) individualized cutting guides were placed on the femoral condyle platform and nails were inserted into the pilot holes to fix the cutting guides. (**B**) double hole drilling. (**C**) the cutting guides were removed, and the prosthesis was manually placed

In the PSI group, UKA was performed according to the following protocols: after a line was drawn on the femoral condyle from top to bottom, individualized cutting guides were placed on the femoral condyle and manually held in place by the surgeon. Two or three nails were inserted into the pilot holes to fix the cutting guides and maintain their stability (Fig. 3A). After double-hole drilling (Fig. 3B), the cutting guides were removed, and the femoral cutting block was drilled along the holes to complete the osteotomy using metal bone cutting tools (Fig. 3C). Finally, the prosthesis was manually placed (Fig. 3C).

In the CI group, the femoral drilling holes were located 1 cm frontal to the anterior edge of the femur. An intramedullary guiding rod was inserted close to the medial wall of the femoral condyle. A drilling guide was placed to insert the intramedullary rod connector (valgus, 7°; posterior slope, 10^o^) into the intramedullary guiding rod to locate and complete the drilling on the femoral condyle, followed by osteotomy via metal bone cutting tools and manual prosthesis placement.

### Evaluation on osteotomy time

Osteotomy time was defined as the amount of time required to accomplish the location, osteotomy, and prosthesis placement. The osteotomy time was routinely recorded. It was used to assess the convenience of PSI in guiding osteotomy.

### Measurements of coronal varus/valgus angle of femoral component

**Fig. 4:**
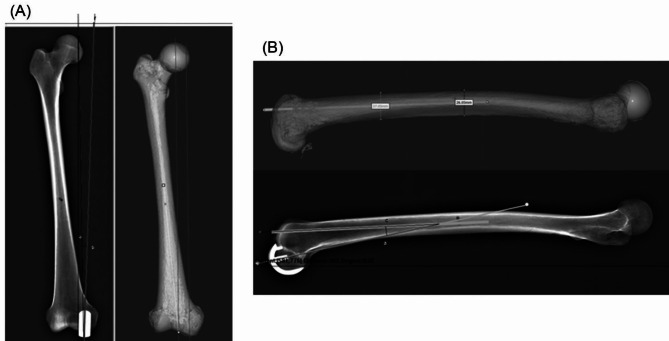
Postoperative coronal and sagittal alignment of the femoral prostheses evaluated using X-ray radiography and measured using e-Ruler software. (**A**) anteroposterior-view radiographs to assess the coronal alignment, which was defined as the angle between the alignment of prosthesis (line a) and the femoral mechanical axis (line b). The left panel shows the radiological outcomes and the right panel shows femoral prostheses. (**B**) lateral view-radiographs to assess the sagittal alignment, which was defined as the angle between the alignment of prosthesis (a) and the anatomical axis (c). The top panel shows femoral prostheses and the bottom panel shows radiological outcomes

The varus/valgus angle was used to evaluate implant alignment on the coronal plane, which was defined as the angle between the femoral mechanical axis and alignment of the prosthesis (Fig. 4A). It was measured postoperatively using the e-Ruler software 1.1 (Softonic International Corp., San Francisco, California, USA). All measurements were determined by three joint surgeons. A deviation ≥ 3° from the designed orientation (0°) was defined as an outlier.

### Measurements of sagittal posterior slope angle of femoral component

The posterior slope, defined as the angle between the alignment of the prosthesis and the anatomical axis, was used to evaluate implant alignment on the sagittal plane (Fig. 4B). It was measured postoperatively by three joint surgeons using Euler software. A deviation ≥ 3° from the designed orientation (10°) was defined as an outlier.

### Measurements of the femoral valgus angle

The femoral valgus angle, defined as the angle between the mechanical and anatomical axes, was used to display the basic information of each specimen (Fig. 1B). The femoral valgus angle was measured preoperatively using the e-Ruler software.

### Statistical analysis

All data were expressed as mean ± standard deviation and analyzed using SPSS 18.0 statistical software (SPSS Inc., Chicago, Illinois, USA). The comparison between the PSI and CI groups was performed using a two-tailed, paired Student’s t-test. The significance level was set at two-tailed p < 0.05.

## Results

### General results

There were no significant differences in age (59.88 ± 9.18 years vs. 57.92 ± 9.28 years, p = 0.414), height (159.84 ± 8.92 mm vs. 162.28 ± 7.77 mm, p = 0.346), weight (52.80 ± 10.10 kg vs. 53.12 ± 8.39 kg, p = 0.883) and femoral valgus angle (5.58 ± 1.23^o^ vs. 5.91 ± 1.05^o^, p = 0.303) of cadaveric specimens between the PSI and CI groups (Table 1), indicating their comparability.

**Table 1:** Comparison of general information of cadaveric specimens between PSI and CI groups

Group	Age (years)	Height (millimeters)	Weight (kilograms)	Femoral valgus angle (^o^)
PSI group (n = 25)	59.88 ± 9.18	159.84 ± 8.92	52.80 ± 10.10	5.58 ± 1.23
CI group (n = 25)	57.92 ± 9.28	162.28 ± 7.77	53.12 ± 8.39	5.91 ± 1.05
Statistic value (t)	0.831	-0.961	-0.149	-1.053
P-value	0.414	0.346	0.883	0.303

PSI, patient specific instrumentation; CI, conventional instrumentation

### Comparison of osteotomy time

Individualized cutting guides were successfully designed for the 25 femoral cadaveric specimens in the PSI group. All cutting guides closely fit the anatomical structure of the femoral condyle, and no obvious displacement was observed after the nails were inserted into the pilot holes. Surgery was successfully performed on all 50 femoral cadaveric specimens. The osteotomy time was found to be significantly shorter in the PSI group than the CI group (3.12 ± 0.65 min vs. 4.33 ± 0.73 min, p < 0.001) (Table 2).

**Table 2:** Comparison of the conveniency and accuracy for positioning of the femoral component of the implants between PSI and CI groups

Variables	Osteotomy time (minutes)	Coronal alignment of prostheses (^o^)	Sagittal alignment of prostheses (^o^)
PSI group (n = 25)	3.12 ± 0.65	1.43 ± 0.93	8.80 ± 0.65
CI group (n = 25)	4.33 ± 0.73	2.65 ± 1.50	6.29 ± 1.88
Statistic value (t)	-12.194	-3.666	6.959
P-value	< 0.001	0.001	< 0.001
Increased (or decreased) fold*	-1.20 ± 0.20	-1.19 ± 1.75	2.50 ± 1.81

PSI, patient specific instrumentation; CI, conventional instrumentation. *An increase or decrease in PSI group relative to the CI group

### Comparison of coronal varus/valgus angle of femoral component between the two groups

In the PSI group, the mean coronal alignment was 1.43 ± 0.93° of varus with two (8%) slight outliers (3.2° and 3.1°). However, in the CI group, the mean coronal alignment was 2.65 ± 1.50° of varus, with nine (36%) outliers in excessive varus (range 3.11°–7.35°) (Table 3). Further, the coronal component angle was significantly different between the PSI and CI groups (1.43 ± 0.93° versus 2.65 ± 1.50°; p = 0.001) (Table 2).

**Table 3:** Raw data of cadaveric specimens in PSI and CI groups

Group	Age (years)	Height (millimeters)	Weight (kilograms)	Femoral valgus angle (^o^)	Osteotomy time (minutes)	Coronal alignment of prostheses (^o^)	Sagittal alignment of prostheses (^o^)
PSI group (n = 25)	56	150	45	5.9	3.5	0.15	9.82
	60	168	75	3.78	2.9	2.82	9.05
	73	175	50	4.92	4.2	0.44	7.82
	56	155	65	4.94	3.6	1.56	8.52
	80	163	45	4.35	3.8	2.51	8.67
	61	162	72	5.61	2.8	1.53	9.42
	66	153	65	3.78	3.2	2.85	9.02
	48	150	47	4.92	2.5	2.73	9.05
	58	160	51	4.94	4.5	0.56	8.73
	46	152	56	4.35	2.7	0.78	8.91
	58	153	46	5.61	2.8	0.9	9.13
	73	158	42	7.9	3.1	0.28	9.5
	60	157	52	5.3	3.5	0.76	9.2
	56	150	45	8.1	3.5	1.25	7.85
	63	165	53	5.88	3.2	1.32	7.92
	56	178	60	4.99	4.1	1.45	8.01
	45	155	46	5.78	2.5	1	8.52
	73	170	40	6.98	3	1.35	8.9
	56	165	65	5.9	3.2	1.65	9
	45	154	38	3.78	3.6	0.32	9.8
	71	158	50	4.92	2.3	1.1	7.8
	55	178	47	6.94	2.4	3.2	7.6
	58	145	65	7.35	3.1	3.1	9.5
	58	158	45	5.61	2	1.5	9.2
	66	164	55	6.9	2.1	0.59	9.1
CI group (n = 25)	45	153	47	4.92	4.5	0.18	5.22
	75	150	56	7.78	3.9	1.12	8.37
	56	160	65	5.35	5.2	3.11	3
	65	152	50	6.82	4.6	1.33	9.84
	65	153	60	5.58	5.8	1.6	5.1
	72	158	75	5.88	3.8	1.8	4.77
	65	157	52	4.99	4.2	5.03	7.72
	57	150	45	5.78	3.5	7.35	5.6
	65	170	51	4.98	5.5	3.75	6.5
	56	158	60	5.9	3.7	2.4	5.3
	56	172	42	6.78	3.8	2.61	3.5
	42	170	57	4.92	4.1	3.75	8.5
	57	158	55	4.94	4.5	4.1	6.7
	65	175	46	4.35	5.5	3.12	7.9
	56	170	55	7.61	4.2	2.5	4
	60	165	49	4.9	5.1	2.6	5.8
	46	168	47	5.3	3.5	3.56	7.5
	60	158	56	8.1	5	1.6	3.5
	65	170	65	6.82	5.1	1.7	7.5
	38	165	42	4.58	3.5	3.5	8.6
	50	166	52	5.88	4.3	2.5	5.6
	47	158	45	6.99	4.1	2.3	4.5
	65	172	45	5.78	4	1.8	6.1
	65	171	65	6.98	3.6	2.5	9.1
	55	158	46	5.9	3.2	0.5	7

PSI, patient specific instrumentation; CI, conventional instrumentation

### Comparison of sagittal posterior slope angle f femoral component between the two groups

The mean posterior slope was 8.80 ± 0.65° in the PSI group and 6.29 ± 1.88° in the CI group, which was statistically significant (p < 0.001). Position deviation from the preoperative plan in the sagittal plane was observed in 15 (60%) implants in the CI group. However, no positioning deviation was observed for implants in the PSI group (Table 3).

## Discussion

### Comparison of the accuracy for positioning the femoral component of the implants between the PSI and CI procedures

Using femur models of dissected cadavers, our study indicated that the PSI system may have the potential to improve the accuracy of positioning femoral implants in UKA. Although the difference was small [15], the coronal component angle of the implants was significantly lower (1.43 ± 0.93° vs. 2.65 ± 1.50°, p = 0.001) and the mean posterior slope was significantly higher (8.80 ± 0.65° vs. 6.29 ± 1.88°, p < 0.001) in the PSI group than in the CI group. More importantly, there were few outliers outside the tolerance interval (± 3° ) for the planned orientation in the PSI group, but nine (36%) and 15 (60%) in the CI group in the coronal and sagittal alignment, respectively. Our conclusions are in line with the study of Jaffry et al. [18] but contradictory to the studies of Alvand et al. [21], Ollivier et al. [22], Sanz-Ruiz et al. [23], and Leenders et al. [24], all of which showed similar accuracies of the PSI and CI procedures with regard to femoral component positioning. These differences may be attributed to the following reasons: (i) a dry bone model was used in our study as well as that by Jaffry et al. [18]; this may not be completely similar to the complex knee status of patients; (ii) in these previous studies, senior surgeons with extensive experience of CI performed the UKA, which resulted in a lower incidence of surgical outliers. However, in our study, PSI and CI were performed by inexperienced surgeons individually guided by experienced faculty members, and were asked to replicate the preoperative plan. Thus, our study reflects the superiority of the PSI procedure. Our hypothesis could be indirectly demonstrated by the studies of Ng et al. [20] and Jones et al., [27] in which novice surgeons were instructed to perform UKA; the results showed that PSI could improve plan adherence, especially compound rotational error of the femoral implants; (iii) MRI was used for preoperative templating and planning of the cutting guides in the studies of Alvand et al. [21], Ollivier et al. [22], Sanz-Ruiz et al. [23], and Leenders et al. [24]. It has been claimed that bone models generated using MR images are dimensionally less accurate than those generated from CT images. In addition, the bone models generated from MR images are visibly inferior to those generated from CT images [28]. This may influence the 3D model construction and final position of the cutting guides, leading to deviations from the actual effects of PSI. CT is currently more favorable because of reduced scanning times, increased availability, and low cost. Therefore, CT-based 3D models were used for UKA in our study.

### Convenience of PSI in guiding osteotomy compared with CI

Another interesting finding was that the osteotomy time was significantly shortened in the PSI group compared with the CI group (3.12 ± 0.65 min vs. 4.33 ± 0.73 min), which confirmed our expectations and was in line with the results obtained from previous studies of TKA [29–31] or UKA surgery [18]. However, there are contradictory results concerning the potential effects of PSI on surgery time reduction in comparison with CI in the study of Alvand et al. [21] [PSI: 75.3 (range, 53.0–90.0) min versus CI: 63.5 (range, 50.0–82.0) min, which may have resulted from the complexity of the knee status of patients and small sample size.

### Limitations

This study has a few limitations. First, this is a cadaver study, and the correlations between femoral component orientation of the implants and long-term follow-up outcomes could not be performed. Second, the sample size is small, with only 25 cadaver bones included in each group, which may have influenced the statistical findings. However, the similar nature of the two populations preoperatively and the randomized design may help mitigate this issue. Third, a deviation > 3° was used to define outliers, although there is only limited clinical evidence for this cutoff level; fourth, although individualized cutting guides improve precision and reduce surgery time, the time required for instrument preparation (including images acquired by CT or MRI, approval of the surgical plan by the surgeons, and manufacturing of the cutting guides) should not be neglected, which may inevitably add costs to the arthroplasty procedure [32]. Thus, further studies are needed to confirm the superiority of PSI in terms of surgical costs.

## Conclusion

This is the first study to use a cadaver model to provide supporting evidence that the femoral implant can be placed at a more accurate position using the customized cutting guide technique during UKA compared with CI. Thus, the PSI technique seems to be a promising strategy to optimize implant positioning for UKAs and, therefore, improve the longevity of implants.

## Data Availability

All data generated or analyzed during this study are included in this article.

## Abbreviations

UKA: Unicompartmental knee arthroplasty
TKA: Total knee arthroplasty
3D: Three-dimensional
CT: Computed tomography
MRI: Magnetic resonance imaging
PSI: Patient-specific instrumentation
CI: Conventional instrumentation
